# A Framework of Vehicular Security and Demand Service Prediction Based on Data Analysis Integrated with Blockchain Approach

**DOI:** 10.3390/s21103314

**Published:** 2021-05-11

**Authors:** Zeinab Shahbazi, Yung-Cheol Byun

**Affiliations:** Department of Computer Engineering, Institute of Information Science Technology, Jeju National University, Jejusi 63243, Korea; zeinab.sh@jejunu.ac.kr

**Keywords:** blockchain, multi-task learning, machine learning, taxi demand service, long short-term memory

## Abstract

The prediction of taxi demand service has become a recently attractive area of research along with large-scale and potential applications in the intelligent transportation system. The demand process is divided into two main parts: Picking-up and dropping-off demand based on passenger habit. Taxi demand prediction is a great concept for drivers and passengers, and is designed platforms for ride-hailing and municipal managers. The majority of research has focused on forecasting the pick-up part of demand service and specifying the interconnection of spatial and temporal correlations. In this study, the main focus is to overcome the access point of non-registered users for having fake transactions using taxi services and predicting taxi demand pick-up and drop-off information. The integration of machine learning techniques and blockchain framework is considered a possible solution for this problem. The blockchain technique was selected as an effective technique for protecting and controlling the real-time system. Historical data analysis was processed by extracting the three higher related sections for the intervening time, namely closeness and trend. Next, the pick-up and drop-off taxi prediction task was processed based on constructing the components of multi-task learning and spatiotemporal feature extraction. The combination of feature embedding performance and Long Short-Term Memory (LSTM) obtain the pick-up and drop-off correlation by fusing the historical data spatiotemporal features. Finally, the taxi demand pick-up and drop-off prediction were processed based on the combination of the external factors. The experimental result is based on a real dataset in Jeju Island, South Korea, to show the proposed system’s efficacy and performance compared with other state-of-art models.

## 1. Introduction

**Figure 1:**
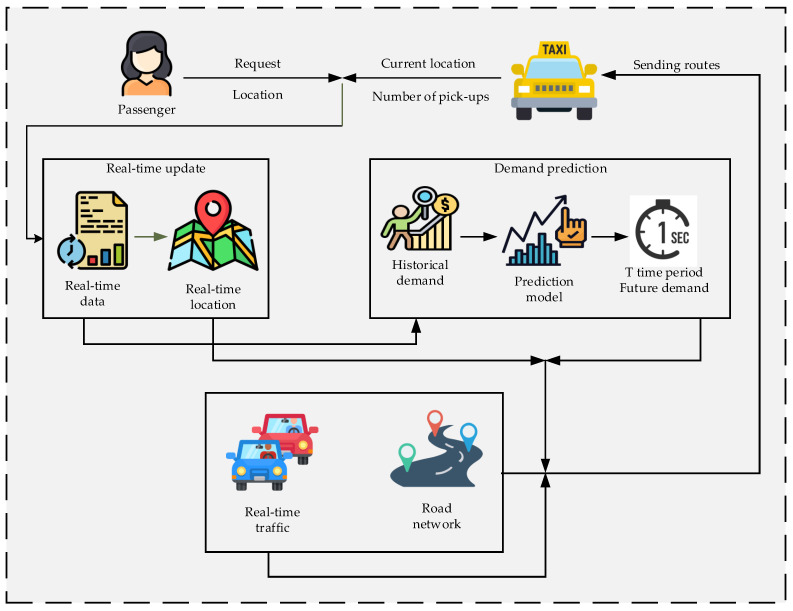
Overview of the regular and ride-hailing taxi services proposed system.

In modern urbanization, the lifestyle of people significantly changes the usage of public transportation, especially taxi services, which is a comfortable and convenient choice for most people travelling when compared with the high costs of using a car and paying for parking lots and other expenses. This is evident in the increase of ride-hailing services in Jeju Island, of which its density and utilization have ineffective resources. Regular taxis in Jeju are not able to obtain the location of a passenger. Conversely, ride-hailing service such as Kakao Taxi, enables a passenger to send a request for a nearby ride-hailing taxi to their selected location. They can use the taxi service easily, comfortably, and it avoids a long waiting time. Based on customer’s experience of using both taxi services, it is important to know that the nearest ride-hailing taxi service might take more time to reach a passenger’s location. In this case, there is a need to improve the utilization and enhancement of the efficiency of both types of taxi services, which is for the benefit of the driver and passenger. The vehicular Social Network (VSN) is the best way to understand the structure of Intelligent Transportation System (ITS) [1]. The general combination of communication technologies and sensors causes the VSN platform to overcome the ITS problems, e.g., such as a traffic anomaly, road density, and other barriers. The floating cars become important components for standing taxis in VSNs [2,3]. Using the mechanism for the system’s security based on the various activities in this domain has the possibility of tracing in real-time, e.g., traffic, weather, damage, the repair of a vehicle, etc. From another point of view, based on the compromising of Internet of Things (IoT) sensors, the intruders can update their points and credits and similarly disable the located place. Any changes in the information or saved data are not visible for the drivers and passengers of the network, making such people more comfortable to conduct such misbehavior [4]. The main usage of blockchain technology is to trace, track, and stand to the relationship between the huge amount of stored data and simplify the contents without the need for the cloud. Figure 1 illustrates the overview of the proposed architecture for regular and ride-hailing taxis. There are three main layers in this system, as shown. The first layer contains the collected real-time data from the passenger and taxi driver. This data includes the passenger location and driver status if it is available or occupied and similarly the completed pick-up numbers. The second layer is the demand prediction layer which performs every period as *T*. The *T* time period shows the demand prediction for only once in every *T* time. Updating the demand considers the number of pick-ups based on the total *T* time period. The third layer contains the matching routes between the passenger and taxi. The route selection is based on real-time traffic information by minimizing the time expected to reach and road network; the request is sent to a nearby taxi, closer to the passenger.

The core of this research is the integration of machine learning and blockchain framework to overcome the transactional problem, reducing the waiting time and travel time of passenger based on the requested location. We have used the Hyperledger Fabric framework to secure the transaction system by giving a specific ID for each user which gives authorization to access the system. Similarly, placing access limitations on a driver and passenger’s private information. In summary, we have proposed a secure platform for taxi demand service, reducing the waiting time of passengers traveling in Jeju island.

The main contribution of this paper is as follows:

- Considering the pick-up and drop-off taxi demand prediction based on the related tasks and creating feature extraction components based on LSTM and multi-task learning;
- Considering the urban taxi demand situation by capturing the spatiotemporal and complex correlation for the pick-up and drop-off process;
- External factors combination and conditions, e.g., weather, daily situation, and transportation conditions for predicting taxi demand;
- Applying blockchain technique gives security and transparency to the system based on the IoT devices between passenger and driver;
- Storing the vehicle information such as car number, driver rating, etc., into the blockchain framework;
- Applying the theoretical analysis on the real-world dataset taken from Jeju Island, South Korea to execute a better performance in comparison to other baselines;
- Reducing the unnecessary distances to improve the accuracy of demand prediction;
- Evaluating the prediction differences errors in one day within the different time period;
- Using the traditional dispatching baselines to get good prediction results and improve performance;
- Overcoming the problem of fake transactions using the Hyperleger Fabric blockchain framework;
- Specifying the valid users to access driver and passenger information.

This paper is divided as follows: Section 2 presents the practical literature review of predicting traffic data. Section 3 presents the steps of the problem explanation. Section 4 presents the pick-up and drop-off prediction of taxi demand services. Section 5 presents the experimental results and design verification, and finally, we summarize this paper in the conclusion.

## 2. Related Work

The field of Machine Learning (ML) is a wide area in different topics and research works, e.g., prediction problems. Vasileva et al. [5], proposed the fast calculation for the parameters of macroscope based on deep neural network construction. The presented system is to find the relationship between stochastic and macroscopic parameters. Feng et al. [6], presented overcoming the stochastic media based on the poroelasticity problem and defect prediction for imbalance prediction issue. Nguyen et al. [7], presented the multiobjective optimization issue based on genetic algorithms through nondominated sorting. Kong et al. [8], presented the prediction of critical transition based on the outstanding problems for the nolinear dynamic problem. In this process, machine training is based on the normal functions with the attractor of chaotic.

Computing services such as machine learning techniques for critical transactions, using the multi-perspective services are used to overcome the higher educations adoption quality and risks, etc. Ali et al. [9], proposed the cloud service based multi-perspective adoption to overcome risks in the higher education system. Dewanta et al. [10] presented the fog computing service for establishing the trust in the vehicular system based on the blockchain framework. Kasemsap et al. [11] presented cloud computing techniques and overview based on the big data and semantic analysis.

A recommendation system such as supporting the customer view in terms of online shopping, educational system, manufacturing design, etc. is considered. In [12], the proposed system is topic modeling of the short text documents based on using deep reinforcement learning. Short text documents contain the issue of lacking information as it is difficult to extract knowledge and the main core of a sentence, this process uses a reinforcement learning algorithm, which is a learning-based algorithm to extract the meaningful parts of the document. In [13], the main core is the focus on a online shopping mall using the XGBoost algorithm to improve the performance of the online system. In [14], the proposed system is the content-based filtering recommendation system to extract useful knowledge for successful recommendations based on user preferences. In [15], the proposed system is tweeter and article recommendation for e-learners. The main core of this system is based on the reinforcement learning algorithm and improving the performance of the online education system. In [16], text segmentation based on the Latent Dirichlet Allocation topic modeling is presented. The process is based on domain-independent unsupervised learning for knowledge discovery from short text docuements. In [17], the proposed system is the automatic knowledege extraction from social media content using a docment classification approach.

In the latest research topic, deep learning has become a popular field in natural language processing, computer vision, etc. [18,19,20]. Moreover, in many types of research, the usage of multi-task learning shows the improvement of application performance. In this section, a brief literature review of the traditional traffic prediction approaches and traffic prediction deep learning approaches are presented.

### 2.1. Traffic Prediction Traditional Approaches

The time-series algorithms are proposed for the first time to predict the Auto Regressive Integrated Moving Average (ARIMA) model. Yang et al. [21] proposed the prediction based on the ARIMA model for urban traffic to improve prediction performance. There is more related research for traffic prediction using the ARIMA model founded in [22]. Pavlyuk et al. [23] proposed highway traffic flow based on conducting various directions for the prediction of short-term traffic flow. Li et al. [24] set up the neural network-based dynamic radial function to combine the inbound and outbound user volume and predict the outbound ridership. Along with the nature of traffic flow between stochastic and nonlinear, the nonlinear data-driven model applied for the forecasting models is proposed with Pushalsky et al. [25]. Tang et al. [26] proposed the no-linear regression model to evaluate different forecasting models by capturing the time-series dataset to improve process performance. The mentioned approaches mainly focus on traffic data temporal correlation. The condition of traffic is based on the adjacent and farther regions. In [27], Multi-View Spatial-Temporal Network (DMVST-Net) is presented for prediction services of taxi demand. The achieved Mean Absolute Error (MAE) was 16%. The prediction results were daily based, which is not the right option for the driver, and it supposes to be hourly, and the generated running time is quite high. In [28,29], the Integer Linear Program (ILP) is proposed, which is based on identifying the real-time shared vehicles and addressing the current position without future demand. The presented approach contains high computational complexity.

### 2.2. Traffic Prediction Deep Learning Approaches

A recent approach to taxi demand prediction is deep learning, which proves the extracted features and effects of the system from the image. Consequently, the traffic demand service and condition in the city predict is based on images using the deep learning techniques, e.g., Convolutional Neural Network (CNN), to predict traffic data. Zhang et al. [30] partitioned the city into small grids to apply CNN to predict traffic speeds through images. In another work [31], they applied the ST-ResNet deep learning approach to forecasting the in-flow and out-flow of traffic, bikes rent, and return information. Their updated research improves their prediction result by using residual neural networks based on parametric-matrix-based and external information mechanisms. The mentioned research work’s main focus is the spatial correlation related to the traffic dataset. To model the temporal correlation, the extraction of CNN gives the fusion features and does not deploy sufficient temporal correlation. Additionally, the positive aspects of Recurrent Neural Network (RNN), the Long Short-Term Memory (LSTM) variant, and Gated Recurrent Unit (GRU) have successful records in the prediction of traffic data. Chen et al. [32] applied the cascaded LSTM where the time domain changes are based on the lateral dimension. The different observation point is based on the vertical dimension to obtain the spatial-temporal correlation for traffic prediction.

### 2.3. Taxi Ride-Sharing Approach

Some of the research works focus on taxi drivers sharing ride information and details. In [33], the framework of data-driven system is simulated based on the simplifying grid map. This process shows the optimization of the cost function for the provided path to driver, e.g., the distance of travel or gasoline consumption. In [34,35], re-balancing the data-driven system proposed for the vehicles across the region, which contains the lack of prediction in the future demand. There are various solutions, such as providing the graph partitioning based on the bipartite graph with minimum complexity. This process can execute into ride-matching a one-to-one issue. In some of the studies in this area, the matching and strategy of competition issue is considered for the ride-hailing system [36]. The observed solutions for the mentioned problem are building the simple grid map without reflecting on predicting the demand, dispatching the taxi, or selecting the route.

### 2.4. Taxi Demand Based on Blockchain Approach

In [37], the intelligent transportation system is based on traffic generation and probability of trust. The traffic scheme is designed based on the optimal routes for assisting the driver and dynamic guidance. The main scenario of this research is to reduce the consumption of fuel and improve travel timing based on reducing road congestion. In [38], handling the demand of energy based on the blockchain network enabled with an Internet of Vehicle (IoV) is presented. This system controls the transactions using the distributed clustering. This system’s simulation results show this approach has a 40.16% improvement in energy conservation performance and 82.06% in transactions. In [39], the blockchain technology explained in detail for the automatic outline selection of charging stations of vehicles. In [40,41], the power supply chain based on a smart grid for the sustainable electrical power and indoor navigation is presented. This process involves a combination of machine learning and blockchain network for a peer-to-peer energy trading approach. In [42], the smart vehicle fueling mechanisem is presented in a blockchain network. In [43,44,45], the transportation system based on an electric vehicle is presented. This development is based on the smart contract centric inference and combination of blockchain and machine learning. Table 1 illustrates a comparison of eight existing studies related to traffic prediction. The main objectives are the used model and the type of approach they applied the temporal and spatial specification in their proposed system and the research work’s main scenario.

In total, comparing the presented approach with other existing work shows that the taxi demand service was analyzed in terms of forecasting and evaluating the travel distance, finding the differences between ride-hailing and ride-sharing, the effectiveness of using online taxi-hailing service, etc. There are various machine learning techniques applied to improve the system’s performance based on considering the temporal and spatial.

## 3. Preliminary

In this section, the preliminaries of this research are presented. There are two main definitions of the process: The description of the trip and related information for an evaluation and description of the region portion and the evaluation information.

**Description 1** **(Trip).** *In this scenario trip is defined as a tuple ($time_{pick}$, $location_{pick}$, $time_{drop}$, $location_{drop}$, ID), where the $time_{pick}$ presents the time of pick-up, $time_{drop}$ presents the time of dropping-off, $location_{pick}$ presents the pick-up location, $location_{drop}$ presents the drop-off location, and ID presents the number of the trip identification.*

**Figure 2:**
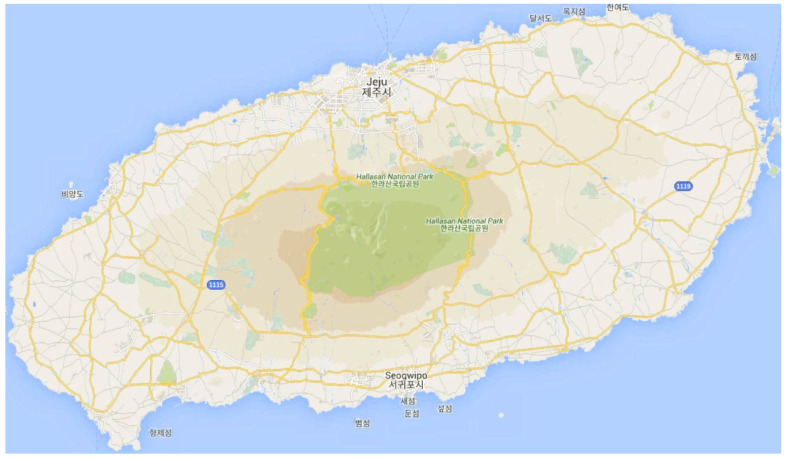
Region partion of Jeju island based on the latitude and longitude into a grid map.

**Description 2** **(Partition of Region).** *The spatial view of this process followed from [54]. As illustrated in Figure 2, the lowest left point of the map considered as *X*, presents the coordinate* $A_{X}\left(lng_{X},lat_{X}\right)$ *, and the top right point of map considered as *Y*, presents the coordinate* $A_{Y}\left(lng_{Y},lat_{Y}\right)$ *. Based on this process, all parts of the city are divided into equal* A∗B *grids. Similarly, the longitude and latitude length is evaluated based on* $\alpha_{lng}$ *and* $\alpha_{lat}$ *where:*

(1) $$A=\frac{lng_{Y}-lng_{X}}{\alpha_{lng}}$$

(2) $$B=\frac{lat_{Y}-lat_{X}}{\alpha_{lat}}$$

*The representation of grid*a,b*is based on the a-th as row and b-th as the column where:*

(3) $$g_{ab}^{lng}\in \left[\alpha_{lng}*a,\alpha_{lng}*\left(a+1\right)\right]$$

(4) $$g_{ab}^{lat}\in \left[\alpha_{lat}*a,\alpha_{lat}*\left(a+1\right)\right],$$

*based on above Equations*a<A*and*b<B.

**Description 3** **(Taxi Demand Service).** *Following the [31,54], to evaluate the determined* a,b *, the taxi demand service pick-up and drop-off based on the time* $T_{j},T_{j+1}$ *estimated as:*

(5) $$c_{j}^{pick,a,b}=\left|\left(trip|t_{pick}\in \left[T_{j},T_{j+1}\right]\wedge location_{pick}\in g_{ab}\right)\right|$$

(6) $$c_{j}^{drop,a,b}=\left|\left(trip|t_{drop}\in \left[T_{j},T_{j+1}\right]\wedge location_{drop}\in g_{ab}\right)\right|.$$

*The time interval*$T_{j},T_{j+1}$*requests for all regions based on the tensor*$C_{j}\in R^{2*a*b}$*, whereby*${\left(C_{j}\right)}_{0,a,b}=c_{j}^{pick,a,b}$*and*${\left(C_{j}\right)}_{1,a,b}=c_{j}^{drop,a,b}$.

## 4. Method

This section presents the various components of taxi demand service. (1) the predictor of taxi demand, (2) the components of taxi-to-region matching, (3) the optimizer of taxi route, and (4) the components of multi-task spatiotemporal feature extraction.

**Figure 3:**
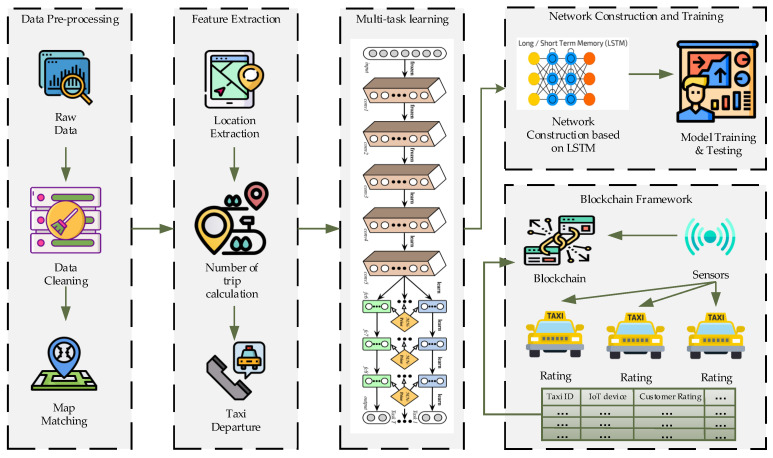
Framework of the proposed system.

Figure 3 presents the overall framework of the proposed system. There is a total of five layers in this system, as shown above. The data pre-processing layer, feature extraction layer, multi-task learning layer, network construction training layer, and blockchain framework layer. All layers are explained in detail in every section.

### 4.1. Predictor of Taxi Demand

**Figure 4:**
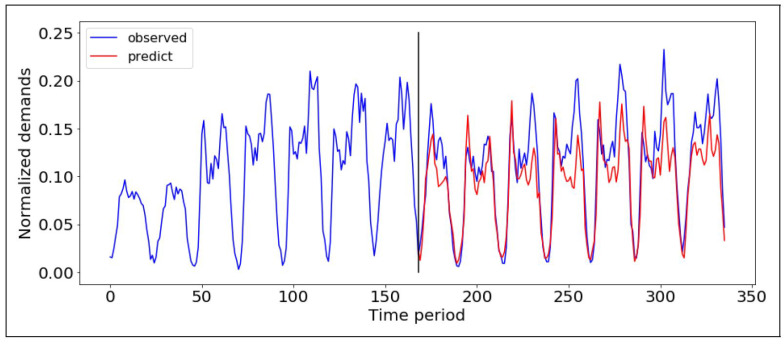
Hourly prediction of the taxi demand based on the LSTM model.

The first step is the prediction of interesting passenger areas. In this process, the LSTM model is applied in the proposed system. In this case, the demand is based on one week, 24 h per day, which is 168 h for predicting the next hour. To do this, the historical data of the previous week applied for predicting the demand for the next hour, which is taken automatically based on weekdays and weekends. This process trends the consecutive features which means the model automatically knows if it is a weekend or weekday. To avoid adding extra information as input, the next hour prediction demands relying on the previous week’s inputs. This means the 168 h of the previous input predicts the demand of next time considering the holidays, weekdays, and weekends as consecutive features. The prediction period of demand is not optional. The matching of the taxi to the region is provided every hour to predict the hourly demand. To predict the traffic demand over time, the main goal is to evaluate each region’s demand and the instant of each period. The demand information and traffic information shared with the help of VSN between the fleet at once, e.g., the remaining passenger’s prediction is evaluated based on considering the picked passengers. Figure 4 shows the performance of the LSTM model based on the hourly prediction in the proposed system. The model trained used the historical data and predicted the future taxi demand. The LSTM model encompasses two hidden layers and one output. The input is the 168 h that is the previous week’s hourly data.

### 4.2. Components of Taxi-to-Region Matching

**Figure 5:**
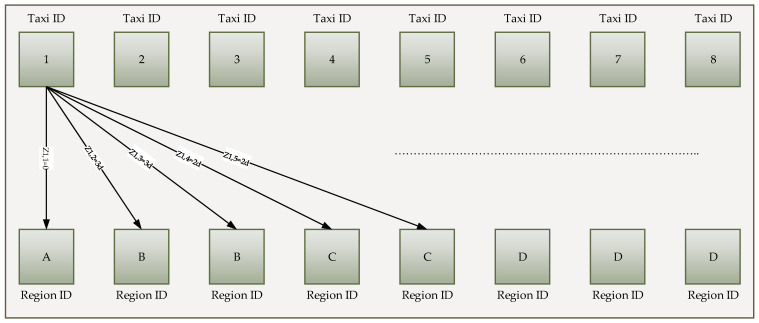
Scenario of the bipatite matching.

After accurate information on taxi demand for each region, the next step is to allocate the taxi’s current location according to the regions. To ensure taxi demand, the first metric is used. If the anticipated demand becomes high, the taxi sends to that location, and it is similar for the opposite location. The taxi’s current location is the second metric that considers minimizing the phase of transaction based on the need for a taxi to reach the decided region. This process reduces the passenger waiting time in case of large-scale problems for a taxi to region, matching the bipartite weighted graph used in Figure 5. The associated weights to the edge of the graph are considered the shortest distances to reach the region’s close border. The highest demand record is duplicated based on each region’s demand in the graph according to multiple times for normalizing the level of demand based on the total number of demand and taxis. As a result, the taxi to region objective is the component matching which means one region is assigned to each taxi. This is considering that the number of taxis are more than the number of regions.

The Taxi ID is *D*, and the Region ID is *E*. As shown in Figure 5 the E=2 and E=3 belong to the same region *B*. The optimization of the matching procedure evaluated based on the ILP problem as below:

(7) $$\left(R\right):Min\sum_{D}\sum_{E}Z_{D,E}X_{D,E}.$$

$X_{D,E}\in 0,1$ subject to:

(8) $$\sum_{E}X_{D,E}=1,\forall D,\;and\;\sum_{D}X_{D,E}=1,\forall E,$$

$Z_{D,E}$ presents the edge of weights linked to the region and the taxi, and $X_{D,E}$ assigns the taxi’s decision for region *D* or *E*. *Z* represents the weight of the edge.

### 4.3. Optimizer of Taxi Route

Road network construction can be done in various ways, e.g., simple techniques of searching and fuzzy logic complex theory [55,56]. This process organized the traffic network based on the complex graph combined with the roads and intersections. Each road *N* contain *M* connections defined as: N∈1,...,M which is segregated into various segments in same length $L_{N}$. The current location in this process is defined as: $\left(I_{n},Ig_{n}\right)$ and the destination is defined as: $\left(J_{n},Jg_{n}\right)$. $I_{n}$ and $J_{n}$ present the street ID and $Ig_{n},Jg_{n}$ presents the segments ID. The route planning problem considers real-time traffic as an optimal solution. Determining the fastest route formulated based on the integer linear programs shows the taxi location and assigned region. This process determines feedback of real-time traffic in the system. ILP also determines the recent data best route and keeps the new data updates. A recurrent Dijkstra algorithm is applied to evaluate the traffic level for every segment to reduce the route optimizer complexity. In the process, routes update every one minute. Procedure one shows the detailed process. Algorithm 1 shows the detailed process of optimizing the taxi route.

**Figure 6:**
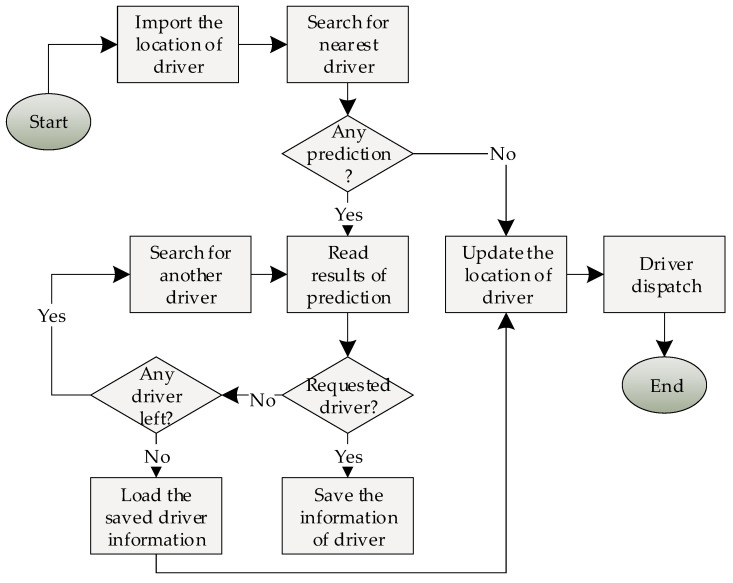
Ride-hailing system flowchart.

Figure 6 presents the ride-hailing system process to take the requested taxi. The first thing is saving the location of the passenger in the ride-hailing system. The second is reading the driver location information to find the closest driver to the requested location. The driver should be free and not reserved for another passenger during working hours.
**Algorithm 1:** Taxi Route Optimizer Procedure.**Require:**$\left(I_{n},Ig_{n}\right)$, $\left(J_{n},Jg_{n}\right)$ and time instant
**while** taxi did not reach to the accepted location **do**
Capture the last update of the procedure based on the available dataUpdate the graph of road networkFind the quick way based on Dijkstra algorithm from $\left(I_{n},Ig_{n}\right)$ and $\left(J_{n},Jg_{n}\right)$Following the suggested routeUpdating the $\left(I_{n},Ig_{n}\right)$**end while**


### 4.4. Components of Multi-Task Spatiotemporal Feature Extraction

In this study, taxi pick-up and drop-off prediction were trained together for each time interval. First, the pick-up features in data and next to the drop-off features extracted. Therefore, feature extraction contains shared information. Moreover, every taxi demand area affects another area. This is the future prediction for taxi demand based on the historical data.

**Figure 7:**
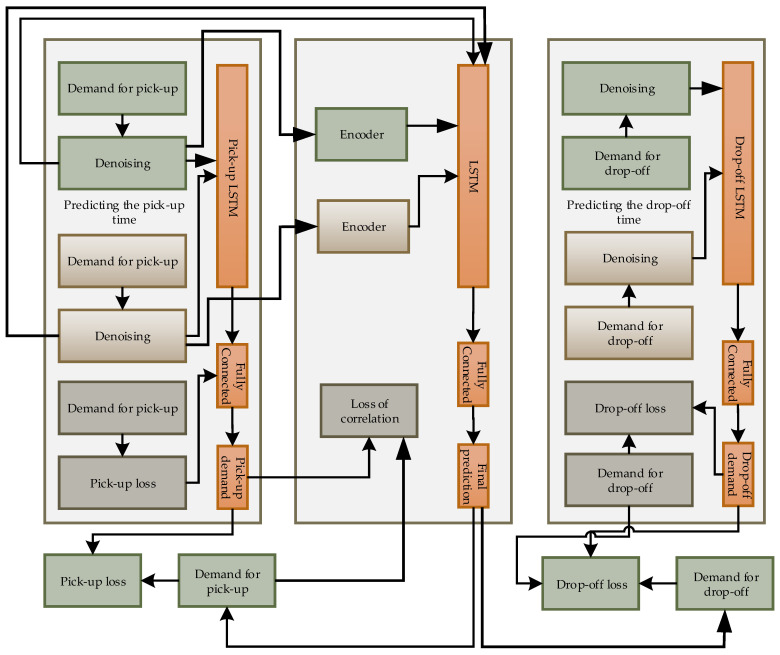
Multi-task learning-based on LSTM model for taxi demand pick-up and drop-off prediction.

Figure 7 shows the multi-task learning architecture based on the LSTM model. There is a total of three layers for taxi demand prediction, which capture the pick-up and drop-off demand based on independent trends. Every single module of prediction has the output and input for any demand.

### 4.5. Blockchain Framework in the Taxi Demand Service

**Figure 8:**
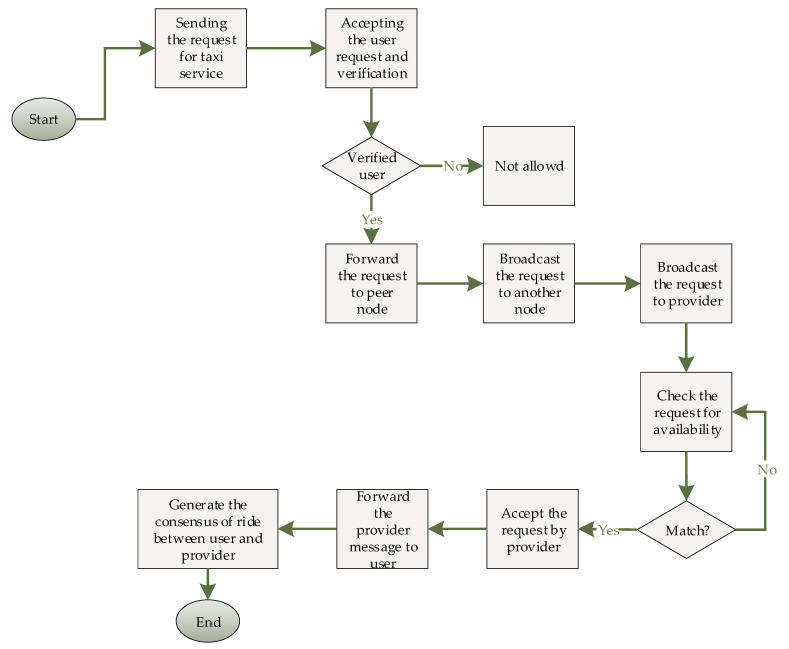
Workflow of the taxi demand blockchain framework.

In this section, the blockchain network in the proposed taxi demand service is explained. The blockchain framework certify the transparency and security between taxi service and passenger. The security process track and traces based on the IoT devices. To provide security for ride-sharing, smart devices track the transmission between the entities. The proposed system contains all the information of registered vehicles and IoT devices. IoT sensors and vehicles’ important information is stored in the database and directly to blockchain framework for tracking all activities. The main reason and necessity of blockchain in this system is to avoid the fake transactional information by hacking other users account and fill their account with others point to use for the payment. The blockchain framework secures the transactions, driver, and passenger profile to block the accessibility of non-member and users without accessibility ID. Figure 8 shows the workflow of the presented system in detail.

## 5. Implementation

In this section, the implementation of the proposed blockchain framework, performance evaluation, simulation results, and transaction information explained in detail.

### 5.1. Performance Evaluation of Blockchain Framework

The proposed blockchain framework for the taxi demand service designed based on the Hyperledger Fabric. Table 2 shows the development environment of the presented system. The used memory for this system is 32 GB with the Ubuntu Linux 18.04.1 LTS operating system. The docker engine and composer were with the version of 18.06.1-ce and 1.13.0. The applied CLI tool is the Composer Rest Server, and the Hyperledger Fabric version is 1.2. There are lots of advantages of Hyperledger Fabric which matches with our proposed approach 1. It needs the permission of membership 2. The level of trust and scalability is high 3. The data basis needs to define and partition for privacy 4. It has the protection for sensitive data and digital keys.

### 5.2. Simulation Results

**Figure 9:**
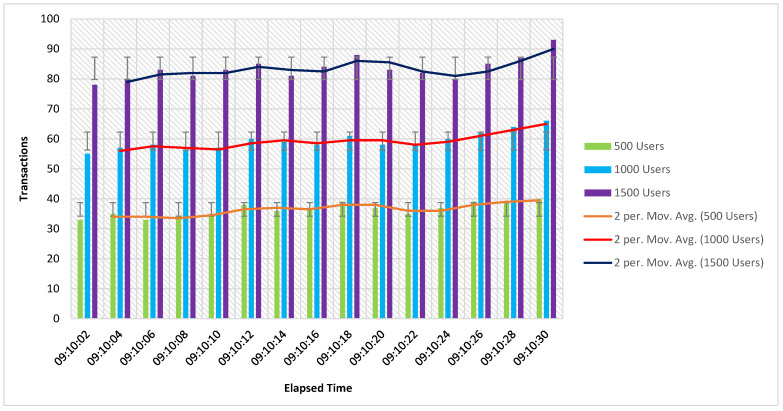
Per second transaction results using Hyperledger Fabric.

Figure 9 shows the transaction results of the Hyperledger Fabric platform per second. We considered three main groups of users for evaluating the performance of the proposed system. Each group contains 500, 1000, and 1500 users. The statistical measurements are used to evaluate the taxi demand service performance in the blockchain framework.

**Figure 10:**
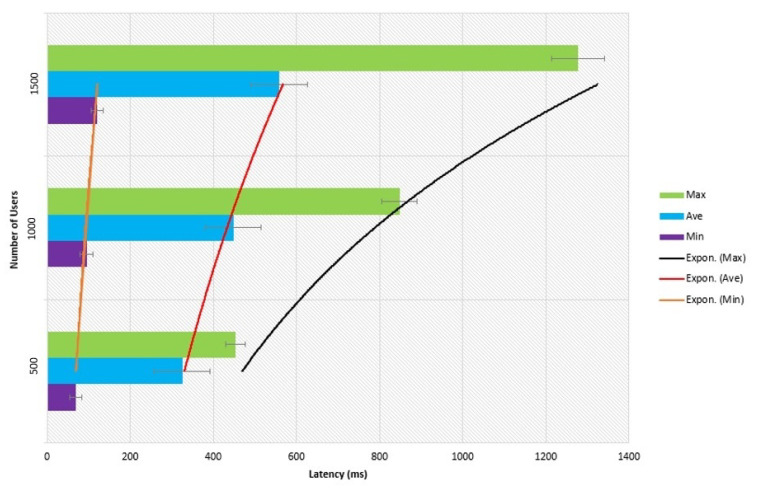
Latency of query transaction.

Figure 10 presents the latency of query transactions in the proposed blockchain-based taxi demand service case study. The defined three groups of users used to access the designed system performance. The system latency takes response time from the designed platform. The transactions are in terms of user groups. As shown in the Figure, the latency is increasing based on maximizing the number of users.

## 6. Results and Discussion

This section contains a brief explanation of the proposed system experimental results and implementation process. There are datasets, baselines, and performance comparisons of various methods, fully covered in this section.

### 6.1. Data

The dataset collected for this research is from the transportation company in Jeju Island. It contains three million records for taxi trips in Jeju from September 2020 to December 2020. The size of the Jeju Island area is 1849 Km^2^. There is a total on average of 72.155 demands containing pick-up and drop-off per day. Each row of data gives the information related to ID, pick-up date and time, drop-off date and time, pick-up longitude, drop-off longitude, pick-up latitude, and drop-off latitude. The external factors define as meteorological features, temporal features, and spatial features. Table 3 shows the data description of the proposed system. Three columns explain the field, definition, and description of the dataset. A total of seven fields were used in the process mentioned: ID, pick-up time, drop-off time, pick-up time longitude, pick-up time latitude, drop-off longitude, and drop-off latitude.

**Figure 11:**
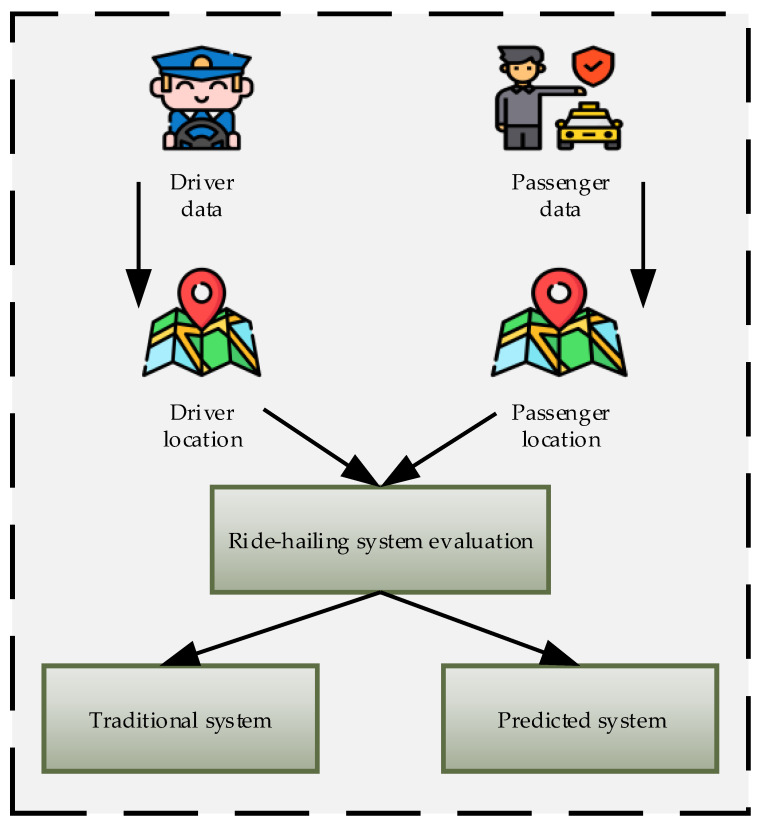
Input and output data diagram.

Figure 11 presents the input and output data process in taxi demand service. There are two types of location information: The driver and passenger location. The result is the prediction of the ride-hailing service to pick-up the passenger and drop-off to the request destination based on the defined machine learning algorithms. Evaluation of ride-hailing system is based on the traditional system and predicted system.

### 6.2. Baselines

The presented approach is trained based on multi-task learning, in other models, the prediction and training of data used for taxi pick-up and drop-off date. The baselines consider Historical Average (HA), Long Short-Term Memory (LSTM), Multiple Layer Perceptron (MLP), and XGBoost. The detailed explanation is as below:

- Historical Average (HA): Based on the previous car pick-up and drop-ff demand average for the location and time of the same place, the prediction value can validate.
- Long Short-Term Memory (LSTM): Is able to learn the long-term dependencies and easily apply for the time-series methods.
- Multiple Layer Perceptron (MLP): The proposed system compared with this approach contains four hidden layers. Respectively, every hidden layer has specific hidden units.
- XGBoost: Is an optimized distributed gradient boosting library that is pliable, portable, and impressive for implementation.

The evaluation metric of this process is the Root Mean Square Error (RMSE) and Mean Absolute Error (MAE) defined as below:

(9) $$RMSE=\sqrt{\frac{1}{N}\sum_{M}{\left(q_{M}-{\hat{q}}_{M}\right)}^{2}}$$

(10) $$MAE=\frac{1}{N}\sum_{M=1}^{N}\left|q_{M}-{\hat{q}}_{M}\right|.$$

The predicted value is defined as ${\hat{q}}_{M}$, the real value defined as $q_{M}$, and the number of totals predicted values defined as *N*.

### 6.3. Performance Evaluation of Predictive Model

The comparison of the presented system with other state-of-art methods is summarized in Table 4. The lowest RMSE belongs to the proposed approach. As shown in the Table, the MLP has the highest RMSE among other approaches. The reason is in this system; the spatiotemporal correlation was not taken into account. The XGBoost algorithm shows acceptable performance, but this method is similar to MAP and does not take the spatial correlation.

**Figure 12:**
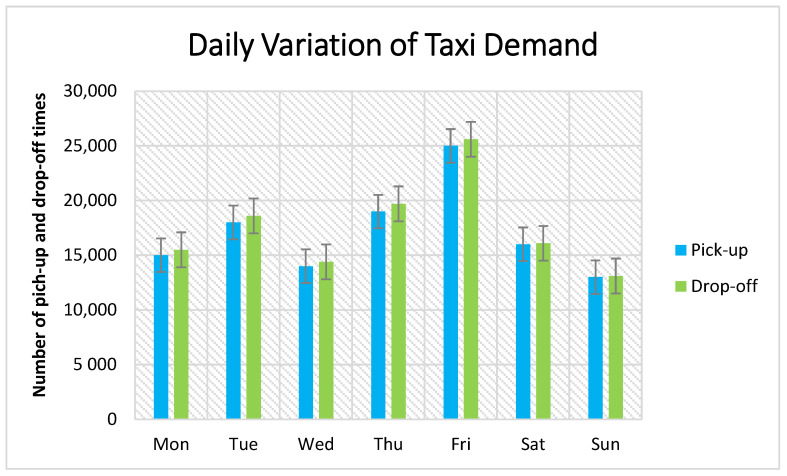
Daily variation of taxi demand.

Figure 12 presents the daily variation of pick-up and drop-off taxi demand in one week from Monday to Sunday. The blue color represents the pick-up records, and the green color represents the drop-off records. As shown in Figure, the highest is on Friday with the maximum pick-up records of 25,000 passengers and almost 26,000 drop-offs. The lowest is on Sunday with a maximum 14,000 of pick-up and drop-off records.

**Figure 13:**
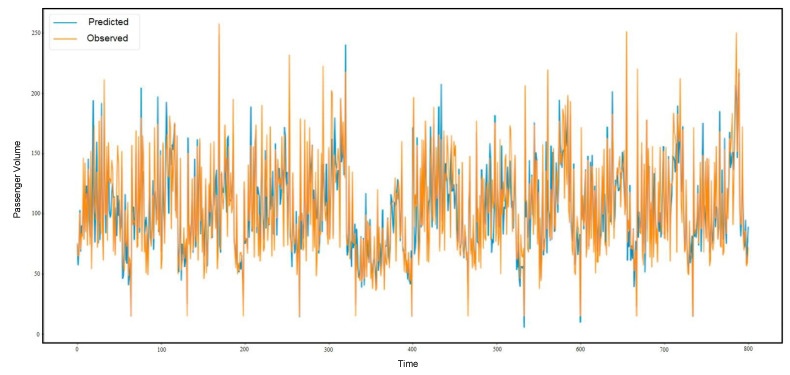
The 15-min prediction result.

**Figure 14:**
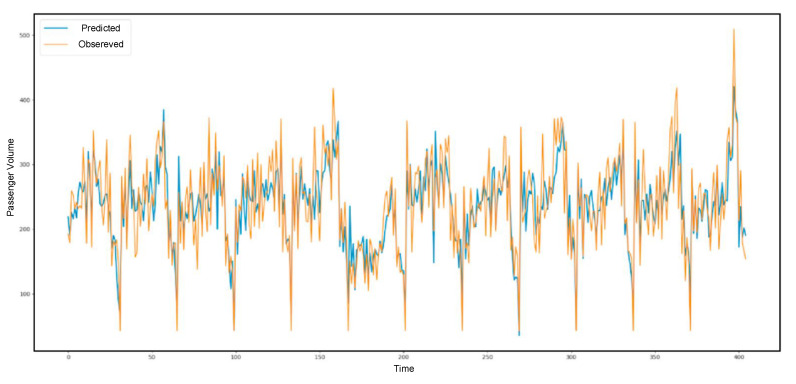
The 30-min prediction result.

**Figure 15:**
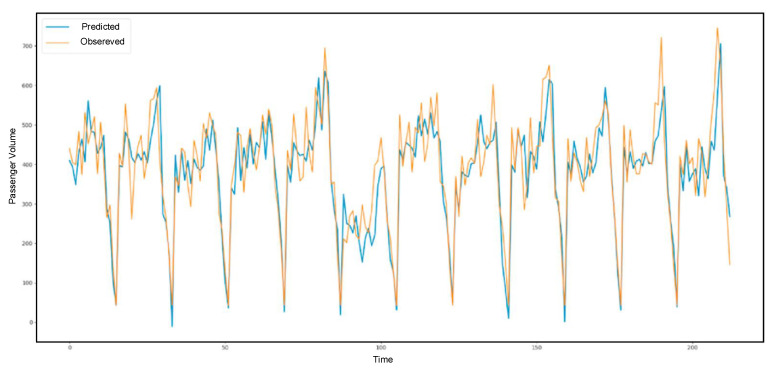
The 60-min prediction result.

Figure 13, Figure 14 and Figure 15 present the prediction result of the taxi demand in three time periods. The first one is within fifteen minutes, the second one is within thirty minutes, and the last one is within sixty minutes. The prediction process is based on time and passenger volume. The predicted records are shown in blue, and the observed records are shown in orange.

Table 5 shows the performance of LSTM based on the determined parameters, epochs, and the number of hidden units to predict the ridership in 15 min. There is a need for settlement between time and performance and select the acceptable parameters for the time and performance set. In this process, the selected parameters are 500 epochs and 100 hidden units.

**Figure 16:**
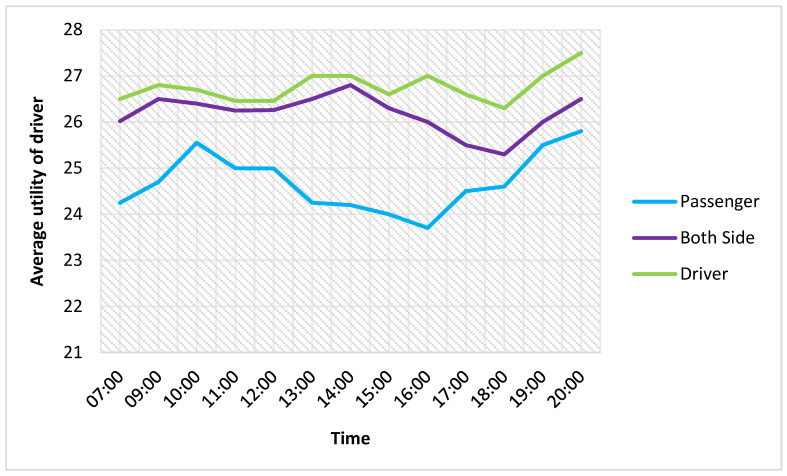
Comparision of the average utility of the driver.

**Figure 17:**
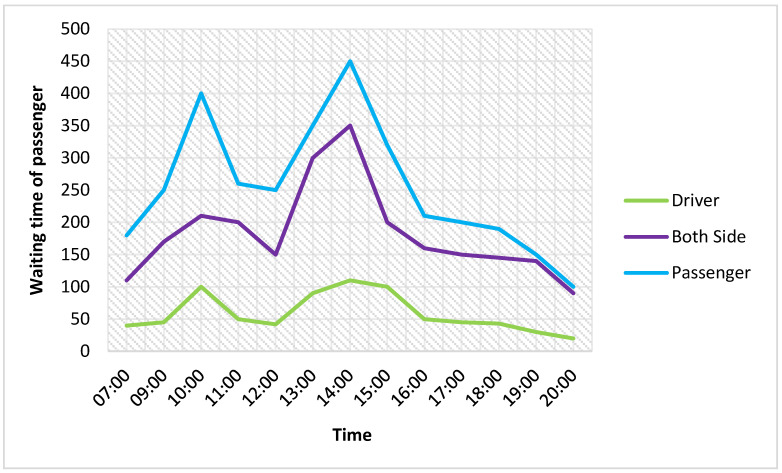
Passenger saved waiting time.

Figure 16 and Figure 17 show the good performance of the proposed method. The average loss of driver utility is about 13% compared with the considered passengers. The saved waiting time of passengers reaches about 70%, which shows the highest saved waiting time.

## 7. Conclusions and Future Work

This research presented the problem of prediction for taxi pick-up and drop-off demand services. We applied the machine learning and multi-task learning approaches to improve this system’s performance and prediction results. Providing transparency and security between the passenger and taxi driver, track- and trace-based on IoT devices and blockchain platform. Information extraction from IoT devices and saving them in blockchain systems gave security and reduced passenger and driver fake actions. The proposed system was tested in Jeju Island, South Korea in taxi demand service, and the result presented a RMSE of 2.22, which shows this system has a lower RMSE in comparison with other baselines. The main contribution of this work is innovating the framework in learning-based for taxi demand. The first step is to extract the spatiotemporal features and apply the LSTM feature embedding to obtain the pick-up and drop-off correlation. The external factors were also considered and finally built the prediction result. This procedure concludes that the standpoint of spatial correlation for taxi demand and set regions will affect the other. From the temporal correlation point of view, the taxi demand is closely similar in a time interval. The spatiotemporal correlation should be together and not separated because the pick-up and drop-off directly affect each other. In another case, the transport conditions, e.g., weather, holiday, etc., also affect the demand for taxis. In future work, we will consider improving the accuracy of prediction based on the time-series algorithms’ performance. Similarly, using the convolutional graph network for predicting taxi demand service and reflect on this model based on the multi-step prediction.

## Figures and Tables

**Table 1:** Existing studies comparison.

Authors	Model	Type	Temporal	Spatial	Main Scenario
Liu et al. [46]	Hybrid	Conv LSTM + Bi-LSTM	Considered	Considered	Freeway
Fu et al. [47]	Deep learning	Sp-LSTM	Considered	Considered	Urban rail transit
Chen et al. [48]	Linear	ARIMA	Considered	Not Cosidered	Urban rail transit
Wu et al. [49]	Hybrid	CLTTP	Considered	Considered	Highway
Liu et al. [50]	SVM	LSSVM	Considered	Not Considered	Urban rail transit
Sun et al. [51]	Hybrid	Wavelet + SVM	Considered	Not Considered	Urban rail transit
Huang et al. [52]	Deep learning	DBN	Considered	Not Considered	Highway
Chen et al. [53]	Deep learning	SAEs	Considered	Considered	Highway

**Table 2:** Development environment of the proposed system.

Component	Description
IDE	Composer-Playground
Memory	32 GB
CPU	Intel(R) Core(TM) i7-8700 @3.20 GHz
Python	3.6.2
Operating System	Ubuntu Linux 18.04.1 LTS
Docker Engine	Version 18.06.1-ce
Docker Composer	Version 1.13.0
Hyperledger Fabric	V1.2
CLI Tool	Composer REST Server
Node	V8.11.4

**Table 3:** Data description.

Field	Definition	Description
ID	id2875421	the taxi id number
Pick-up time	2020.09.14	the passenger pick-up time
Drop-off time	2020.09.14	the passenger drop-off time
Pick-up longitude	−73.982155	the pick-up point longitude
Pick-up latitude	40.767937	the pick-up point latitude
Drop-off longitude	−73.964630	the drop-off longitude
Drop-off latitude	40.765602	the drop-off latitude

**Table 4:** Comparison of various methods performance.

Method	RMSE		
	Pick-Up	Drop-Off	Total
MLP	3.70	4.37	4.20
LSTM	2.65	3.08	3.00
XGBoost	2.47	2.74	2.73
HA	2.77	3.21	3.11
Proposed Approach	2.13	2.32	2.22

**Table 5:** LSTM performance for 15-min prediction.

Prediction Per 15 min		
**Epochs**	**Hidden Unit**	**Required Time (S)**
200	10	33.577
	100	116.153
	500	465.725
500	10	113.616
	100	140.862
	500	1273.240
1000	10	124.501
	100	1851.472
	500	3685.741

## Data Availability

No data available.
